# Analysis of Predistortion Techniques on Fresnel Zone Plates in Ultrasound Applications

**DOI:** 10.3390/s21155066

**Published:** 2021-07-27

**Authors:** José Miguel Fuster, Sergio Pérez-López, Francisco Belmar, Pilar Candelas

**Affiliations:** Centro de Tecnologías Físicas, Universitat Politècnica de València, 46022 València, Spain; jfuster@dcom.upv.es (J.M.F.); serpelo1@teleco.upv.es (S.P.-L.); fbelmar@fis.upv.es (F.B.)

**Keywords:** Fresnel Zone Plate, acoustic focusing, acoustic lens, focusing profile

## Abstract

In this work, we analyze the effect of predistortion techniques on the focusing profile of Fresnel Zone Plates (FZPs) in ultrasound applications. This novel predistortion method is based on either increasing or decreasing the width of some of the FZP Fresnel rings by a certain amount. We investigate how the magnitude of the predistortion, as well as the number and location of the predistorted rings, influences the lens focusing profile. This focusing profile can be affected in different ways depending on the area of the lens where the predistortion is applied. It is shown that when the inner area of the lens, closer to its center, is predistorted, this technique allows the control of the focal depth at the main focus. However, when the predistortion is applied to an area farther from the center of the lens, the acoustic intensity distribution among the main focus and the closest adjacent secondary foci can be tailored at a certain degree. This predistortion technique shows great potential and can be used to control, modify and shape the FZP focusing profile in both industrial and therapeutic applications.

## 1. Introduction

Acoustic focusing is a hot topic in a wide range of industrial [1] and medical applications [2]. One of the newest alternatives devised to focus acoustic waves are acoustic metasurfaces, which can be implemented using different types of structures, such as coiling-up space channels [3,4,5], Helmholtz resonators [6], or subwavelength slits [7]. In recent years, researchers developed a new kind of holographic device based on 3D printed structures capable of focusing complex pressure waves with arbitrary shapes [8,9,10,11]. One of the main drawbacks of these types of structures is that they require a complete 3D design of each one of their unit cells in the case of metasurfaces and a complex 3D propagation and back-propagation optimization algorithm to obtain the desired holographic plate in the case of acoustic holograms. Other interesting alternatives to focus ultrasound waves include spheres [12] or cylinders [13] filled with different liquids, which are able to produce acoustic jets with lateral resolutions beyond the diffraction limit. However, these kind of structures can only focus in very close range applications, which limits their potential.

In this sense, Fresnel Zone Plates (FZPs) are a much simpler yet flexible and powerful alternative to focus acoustic waves. FZPs are monofocal planar lenses employed in a wide range of fields, ranging from optical [14], to microwave [15], and acoustic [16,17,18] applications. This kind of lens consist of a series of concentric rings with decreasing widths, known as Fresnel regions, which can be implemented by either alternating pressure blocking regions with transparent regions (Soret FZPs) [16,18], or phase-reversal regions with transparent regions [19]. In addition, in recent years, a novel kind of lense based on applying a binary sequence to the different Fresnel regions of a conventional FZP [20] has been presented, increasing the versatility of this kind of devices. If Fibonacci or M-bonacci binary sequences are employed, the resulting lens achieves a bifocal focusing profile [21,22,23], while if fractal Cantor binary sequences are used, multifocal focusing profiles with interesting self-similarity properties are achieved [24,25,26,27]. Analogously, binary Thue–Morse sequences provide focusing profiles with bifocal fractal properties [28,29].

It is well known that FZP focusing profiles are sensitive to fabrication errors during the manufacturing stage. In this work, we investigate the effect of introducing controlled errors in the design of the Fresnel rings. We refer to this novel technique as predistortion, due to the fact that the FZP layout is willfully distorted in advance to achieve a certain effect on its focusing profile. These intentional errors, when produced in certain patterns, can be used to modify the shape of the FZP focusing profile in a desirable way. The main parameters of the predistortion technique are the number and location of the Fresnel regions where the predistortion is introduced, as well as the degree of the induced predistortion.

In this paper, we will show that the lens focusing profiles produced by our predistortion technique present certain similarities with the focusing profiles obtained when using Fractal zone plates [24,25,26,27]. The main advantage of our technique is its degree of flexibility, which allows the controling of the focusing profile by modifying either the number of predistortion regions where predistortion is introduced or the severity of the predistortion. Therefore, our technique can be used in all those applications where Fractal zone plates have been successfully applied [25,30,31,32]. Section 4 further develops on the different potential applications of the predistortion technique.

## 2. Predistortion Procedure

**Figure 1:**
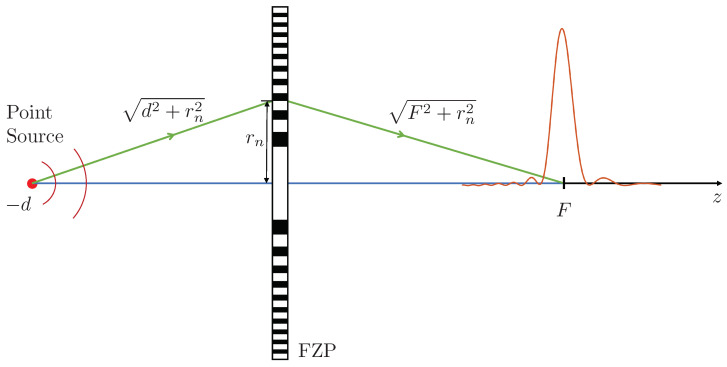
Schematic of FZP focusing with source point excitation.

The governing equation used to design an FZP with point source excitation is given by

(1) $$d+F+n\frac{\lambda }{2}=\sqrt{d^{2}+r_{n}^{2}}+\sqrt{F^{2}+r_{n}^{2}},$$

with n=1,2,…,N, being *N* the number of Fresnel regions, *F* the FZP desired focal length, λ the operating wavelength, *d* the separation between the point source and the FZP, and $r_{n}$ the radius of each Fresnel region. This equation is easily derived from the schematic shown in Figure 1. The Fresnel zone construction principle [16] establishes that the path difference between the direct path (blue path) and the diffracted path through a Fresnel zone (green path) must be an integer multiple of λ/2.

The Fresnel region radii can be directly obtained solving Equation (1) as

(2) $$r_{n}=\sqrt{\frac{{\left[{\left(d+F+n\frac{\lambda }{2}\right)}^{2}-d^{2}-F^{2}\right]}^{2}-4d^{2}F^{2}}{2\left(d+F+n\frac{\lambda }{2}\right)}}$$

If some of the Fresnel region radii obtained from Equation (2) are slightly modified, either by increasing or decreasing their values, an intentional distortion is introduced during the design process of the FZP. This predistortion procedure affects the FZP focusing profile, resulting in some interesting effects. A δ predistortion parameter, ranging from 0 to 1, is introduced in order to characterize the predistortion procedure. This δ parameter is used to indicate the variation of the Fresnel radii, and thus the amount of introduced predistortion. The minimum (non-existent) predistortion corresponds to δ=0, whereas δ=1 corresponds to the maximum (total) predistortion case.

The δ parameter can be mathematically defined using the $\xi_{n}$ parameter previously introduced in [20,24]. For the distortion-free conventional FZP, the $\xi_{n}$ parameter takes the following values:

(3) $$\xi_{n}=\frac{n}{N},$$

and Equation (2) can then be expressed as

(4) $$r_{n}=\sqrt{\frac{{\left[{\left(d+F+N\xi_{n}\frac{\lambda }{2}\right)}^{2}-d^{2}-F^{2}\right]}^{2}-4d^{2}F^{2}}{2\left(d+F+N\xi_{n}\frac{\lambda }{2}\right)}}$$

When a certain amount of controlled distortion is introduced in the design of the FZP lens, the $\xi_{n}$ parameter is also distorted and denoted by $\xi_{n}^{D}$. Odd Fresnel regions (n=2k+1, with $k=0,1,\ldots ,\frac{N-1}{2}$) increase the value of their $\xi_{n}$ parameter, while those Fresnel regions corresponding to even numbers (n=2k, with $k=0,1,\ldots ,\frac{N-1}{2}$) diminish their $\xi_{n}$ parameter values. The corresponding equations are

(5) $$\begin{array}{ccc} \xi_{2k+1}^{D} & = & \xi_{2k+1}+\delta \frac{1}{2N} \end{array}$$

(6) $$\begin{array}{ccc} \xi_{2k}^{D} & = & \xi_{2k}-\delta \frac{1}{2N} \end{array}$$

Not every Fresnel radius needs to be predistorted. Predistortion can be limited to a certain area inside the FZP layout. In this paper, we analyze the effect of applying predistortion to both the inner Fresnel regions and the intermediate Fresnel regions of the FZP. Substituting the distorted $\xi_{n}^{D}$ values given by Equations (5) and (6) into Equation (4), the required predistorted Fresnel radii can be calculated.

In this work, Soret FZP lenses, where the Fresnel regions are either opaque or transparent, are employed. Moreover, the central Fresnel region is considered to be opaque. However, we have carried out additional simulations, and the results presented in this paper are also valid for both Soret FZP lenses with a central transparent region and phase-reversal FZPs with transparent and phase-reversal Fresnel regions.

When the predistortion δ parameter is augmented, Fresnel opaque regions increase their sizes, while Fresnel transparent regions reduce them. This is due to the fact that odd Fresnel radii increase their values whereas even Fresnel radii diminish theirs. If the central Fresnel region is opaque as considered in this analysis, any opaque region in the FZP lens begins at an odd Fresnel radius and ends at the next even Fresnel radius, and the opposite is true for any transparent region. A curious phenomenon takes place when maximum distortion is considered (δ=1). In this extreme case, Fresnel transparent regions disappear in the predistorted area, and therefore, Fresnel opaque regions cover the whole predistorted area, becoming a large whole opaque area. For instance, the fifth Fresnel radius (n=5) corresponds to the end of the third Fresnel opaque region and the beginning of the third transparent region, while the next radius (n=6) corresponds to the end of the third Fresnel transparent region and the beginning of the fourth Fresnel opaque region. When δ=1, the distorted $\xi_{n}^{D}$ parameters for n=5 and n=6 can be calculated as $\xi_{5}^{D}=\frac{5}{N}+\frac{1}{2N}=\frac{11}{2N}$ and $\xi_{6}^{D}=\frac{6}{N}-\frac{1}{2N}=\frac{11}{2N}$, respectively. Both values are equal, which results in the disappearance of the third transparent Fresnel region, while the third opaque Fresnel region ends at the same radius where the fourth opaque Fresnel region begins without any discontinuity, becoming a larger opaque region. A very similar structure involving large inner opaque areas was previously proposed in [33]. In this work, the FZP was modified by placing a pupil device in front of the FZP lens. As it will be discussed later in Section 4, the effect that is achieved on the predistorted FZP focusing profile for the case of maximum predistortion (δ=1) being very close to that experimentally demonstrated in this previous work.

**Figure 2:**
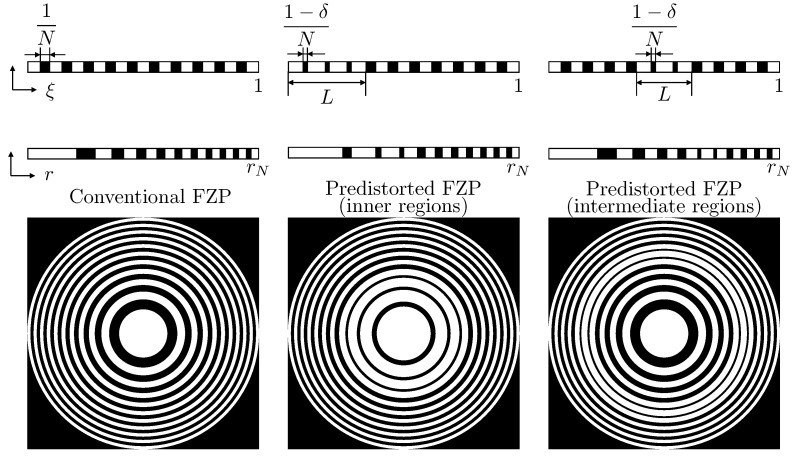
Schematic view of a conventional FZP (**left**), a predistorted FZP with L=7 inner regions (**center**) and a predistorted FZP with L=7 intermediate regions (**right**). Top row represents the different Fresnel radii in the normalized ξ domain, second row shows the Fresnel radii along the *r*-axis, and bottom row represents the corresponding FZP layouts.

Once an FZP is designed, the main parameters that must be considered to apply the predistortion technique are the number of Fresnel regions that are going to be predistorted (*L*), their location, and the degree of the predistortion (δ). In this paper, the location of the predistorted regions is going to be limited to two different situations: inner regions and intermediate regions. Inner regions refer to the Fresnel regions closer to the central opaque region, whereas intermediate regions refer to the Fresnel regions closer to the middle Fresnel region. The application of this predistortion technique is shown in Figure 2, which depicts the Fresnel radii along the normalized ξ domain (top row), the Fresnel radii along the *r*-axis (second row), and the corresponding FZP layouts (bottom row) for three cases: the conventional non-predistorted FZP (left column), the predistorted FZP whith inner regions (central column) and the predistorted FZP whith intermediate regions (right column). As can be observed from the top row of the figure, when the predistortion parameter δ becomes higher, the predistorted transparent regions become smaller, their widths being $\frac{1-\delta }{N}$ when depicted against the normalized ξ domain. The layouts shown in Figure 2 correspond to δ=0.5. When maximum predistortion is applied (δ=1), this width becomes equal to 0 and the predistorted transparent regions totally disappear.

Thus, when considering predistorting inner regions, if for instance L=7 (central column of Figure 2), it results that the first seven regions, starting with the central opaque region (region 1), are going to be predistorted. Therefore, the first four opaque regions and the first three transparent regions become predistorted in this case. On the other hand, Figure 2 (right column) shows the situation when intermediate regions are predistorted. For instance, if N=21, the middle Fresnel region is region 11, and if L=5, the predistorted regions would be in this case Fresnel regions 9,10,11,12 and 13.

## 3. Results

### 3.1. Predistortion Applied to Inner Regions

**Figure 3:**
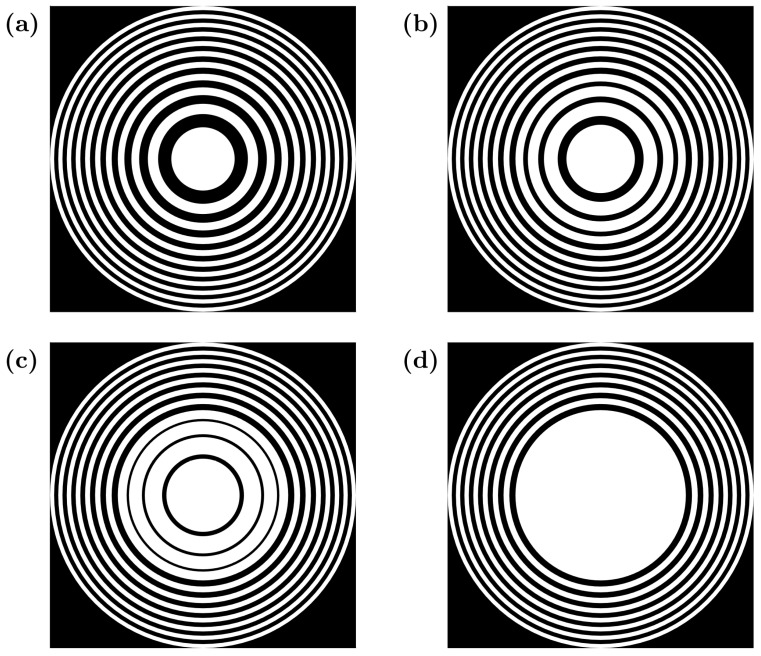
FZP layouts for different predistortion parameters: (**a**) δ=0, (**b**) δ=1/3, (**c**) δ=2/3 and (**d**) δ=1. Predistortion is applied to L=7 inner Fresnel regions.

Figure 3a shows a conventional FZP with N=21, F=50 mm, λ=1.5 mm and d=350 mm. This FZP presents no predistortion and thus its corresponding distortion parameter is δ=0. Figure 3b–d show FZPs with a different degree of predistortion, corresponding to δ=1/3, δ=2/3 and δ=1, respectively. In all cases, the first L=7 inner Fresnel regions have been predistorted. As expected from the analytical model, in the maximum distortion case, corresponding to δ=1 and shown in Figure 3d, Fresnel transparent regions have completely disappeared, whereas the first four Fresnel opaque regions have been fused together, forming a large opaque inner area.

**Figure 4:**
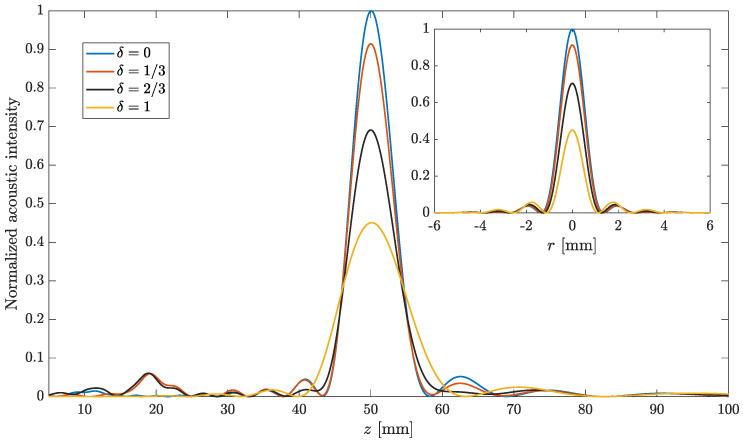
FZP axial focusing profiles for different predistortion parameters: δ=0 (blue line), δ=1/3 (red line), δ=2/3 (black line) and δ=1 (yellow line). The inset represents the corresponding lateral intensity profiles at z=F. Predistortion is applied to L=7 inner regions.

Figure 4 shows the focusing profiles corresponding to the lenses depicted in Figure 3. The main figure shows the FZP axial focusing profile against the *z* coordinate (perpendicular to the plane of the lens), whereas the inset shows the FZP lateral intensity profile at z=F against *r* (parallel to the plane of the lens). In both cases, axial focusing profiles and lateral intensity profiles, the normalized acoustic intensity has been obtained from the square of the acoustic pressure, which has been computed using Equation (7), as shown in Section 6. As the FZP presents axial symmetry, so does its corresponding lateral intensity profile at z=F. Thus, the combination of these two profiles (axial and lateral) gives a good understanding of the performance of the predistorted FZP lens. As can be observed from the figure, when predistortion is increased, the focusing profile is modified in several ways. First, the acoustic intensity peak at the focus diminishes. This can be observed in both the main figure and the inset. The acoustic intensity profiles have been normalized to the case of zero distortion, conventional FZP, for comparison purposes. On the other hand, the focal depth becomes significantly wider (main figure), modifying the shape of the focusing profile. This effect can be very interesting in those industrial or medical applications where a larger area has to be affected. Finally, the inset shows that the focal width is not significantly modified by the induced predistortion.

**Figure 5:**
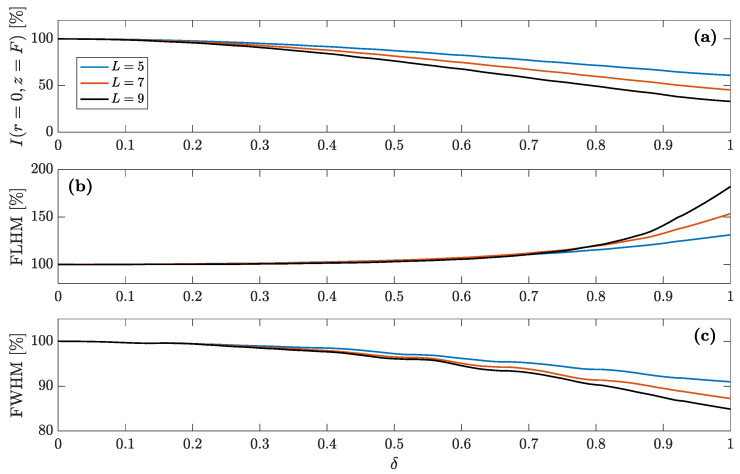
Effects of predistortion on: (**a**) focal intensity peak, (**b**) focal depth, and (**c**) lateral resolution. The L parameter stands for the number of inner regions where predistortion is applied.

In order to get a better understanding of the effect of predistortion on these parameters, Figure 5a–c depict the focal intensity peak, the Full Length Half Maximum (FLHM), and the Full Width Half Maximum (FWHM), respectively, against the distortion parameter (δ) for different values of *L*. The FLHM corresponds to the “Length” of the focus in the axial direction and is calculated from the difference between the *z* locations, at opposite sides of the focus central point, at which the normalized intensity is half of its maximum value. Similarly, the FWHM corresponds to the “Width” of the focus in the radial direction and is calculated as the difference between the *r* locations, at opposite sides of the focus central location, at which the normalized intensity is half of its maximum value. As can be observed from Figure 5b, when the number of predistorted Fresnel regions is L=9, an 80% increment on the focal depth (FLHM) can be achieved for the maximum distortion case (δ=1). On the contrary, the effect of the predistortion parameter on the lateral resolution is less significant (FWHM). A 15% decrease in FWHM can be observed at the most distorted case (L=9 and δ=1). However, the focal intensity is significantly reduced, around 60% for L=9 and δ=1. Therefore, predistortion on the inner Fresnel regions can be used to increase the desired focal depth at the expense of losing some peak intensity at the focus, with a slight enhancement in lateral resolution.

### 3.2. Predistortion Applied to Intermediate Regions

The location of the predistorted Fresnel regions has a significant impact on the shaping of the FZP focusing profile. Thus, when the predistorted regions are shifted from the inner regions to the intermediate regions, the effect of the predistortion on the FZP focusing profile is dramatically different.

**Figure 6:**
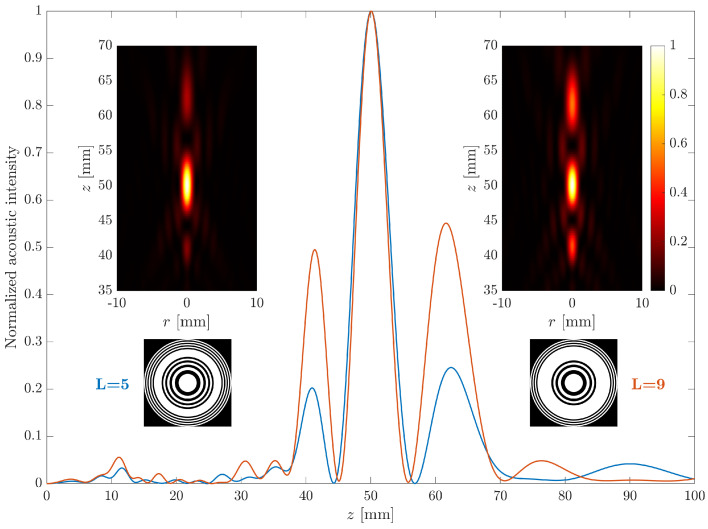
Normalized focusing profiles for L=5 (blue) and L=9 (red). The insets represent the corresponding intensity maps (**top**) and the layouts (**bottom**) of both lenses.

Figure 6 shows the FZP layouts (bottom insets) for two lenses with predistortion applied to the intermediate Fresnel regions, with δ=1 and different *L* values. The L=5 case is depicted in blue and corresponds to the left insets, whereas the L=9 case is depicted in red and corresponds to the right insets. The main figure shows the axial focusing profiles for both FZP lenses. Again, the normalized acoustic intensity has been obtained from the square of the acoustic pressure and Equation (7). As can be observed from the main figure, the FZP focusing profile in this case (predistortion applied to the intermediate regions) is significantly modified compared to the previous case (predistortion applied to the inner regions). Thus, the focusing profile is now very different, introducing two new secondary focal regions, adjacent to the main focus, which is still located at the same focal length as in the conventional FZP case. Depending on the value of the *L* parameter, the weight intensity distribution among the three foci varies. When *L* presents a larger value (L=9), the adjacent foci increase their weights with respect to that of the main focus. The acoustic profiles have been normalized to their maximum values in order to ease their comparison. The insets at the top of Figure 6 show the intensity maps for both cases (L=5 and L=9), against the axial (*z*) and radial (*r*) coordinates.

**Figure 7:**
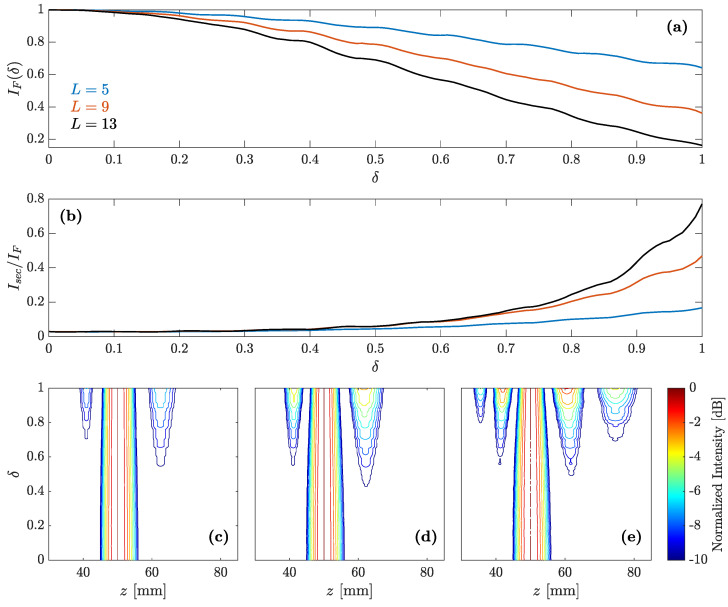
(**a**) Intensity of the main focus normalized to the non-predistortion case. (**b**) Intensity of the higher secondary focus normalized to the focal intensity. Both (**a**,**b**) are depicted as functions of δ for L=5, L=9, and L=13. (**c**–**e**) Focusing profile contour maps as a function of δ for L=5 (**c**), L=9 (**d**) and L=13 (**e**).

In order to get a deeper insight of the predistortion applied to the intermediate regions, Figure 7b depicts the ratio between the acoustic intensities of the larger secondary foci and the main foci as a function of the predistortion parameter (δ), and for three different *L* values. As can be observed from the figure, the intensity distribution among the different foci can change dramatically with the degree of predistortion. The higher secondary focus can become as large as an 80% of the main focus. Thus, in the case of intermediate regions, the predistortion parameter controls the intensity distribution among the different foci of the focusing profile. Figure 7c–e show the focusing profile contour maps against the predistortion parameter for L=5, L=9, and L=13, respectively. All contour maps have been normalized and represented in a logarithmic scale to ease the visualization of the simulation results and their comparison. As can be observed from Figure 7b–d, when the degree of predistortion is increased, a couple of adjacent foci appear in all cases next to the main focus. These secondary foci become stronger (higher intensity) for larger δ values. In the L=13 case, which corresponds to Figure 7d, it can be observed that the predistortion becomes so severe than additional lateral foci have been excited. Therefore, the use of the predistortion technique applied to the intermediate Fresnel regions allows the excitation of secondary foci at fixed locations, with a certain control over the weight distribution of acoustic intensities among the different foci, depending on the degree of predistortion given by the δ and *L* parameters. However, the intensity peak of the main focus is reduced as can be observed from Figure 7a, where the main focus peak intensity is depicted against the predistortion parameter δ. The stronger the predistortion becomes, due to either a larger number of predistorted Fresnel regions (*L*) involved or a higher degree of predistorsion (δ), the lower the main focus intensity level holds out.

## 4. Discussion

In this paper we have shown that the focusing profile of a conventional FZP lens can be modified by predistorting the Fresnel regions. If the inner Fresnel regions are predistorted, it is possible to control the focal depth of the focus by modifying the predistortion value δ without changing the location of the main focus. When the predistorted Fresnel regions are shifted to intermediate positions, several secondary foci appear adjacent to the main focus. The level of these secondary foci can be adjusted using both the *L* and δ parameters. Thus, when predistortion is applied to intermediate regions, we can change the balance between the main and the secondary foci intensities. It is true that these types of focusing profile are not novel and can be achieved using alternative techniques such as Cantor [24,25,26,27] or Thue–Morse [28] binary sequences, but we present our technique as an alternative method with a higher degree of flexibility. In our technique, the *L* and δ parameters allow one to finely adjust the degree of balance among the different foci, whereas this balance is fixed when using the binary sequence techniques. Therefore, all the applications in which the Cantor or Thue–Morse binary sequences have been successfully applied can also be approached with our predistortion technique, but as stated above, with a greater degree of flexibility. In this sense, multifocal Cantor fractal zone plates, which provide similar focusing profiles to those of a predistorted FZP at the intermediate regions, have been recently proposed as optical tweezers with multiple trapping planes [30,31]. Analogously, predistorted FZPs have potential applications as acoustical tweezers for particle trapping and manipulation in many biological and industrial fields [34,35,36,37,38].

Although it is not shown in the paper, we have carried out additional simulations that show that these results can be totally translated from the acoustic domain into the optical domain, where FZPs are also a hot research topic. As stated above, the FZP focusing profile that is obtained when applying the predistortion technique for intermediate regions presents certain similarities with that obtained when Cantor or Thue–Morse binary sequences are used. It has been shown that this type of focusing profile has promising applications in the optical field in reducing the chromatic aberration and extending the depth of field [25,28]. Fractal zone plates based on Cantor binary sequences have also been successfully applied as multifocal intraocular lenses [32]. Therefore, our predistortion technique is also suitable for this type of application, with the advantage of providing a higher degree of flexibility and control over the focusing profile.

In order to experimentally validate the analytical model and the simulations shown in this paper, we rely on some previous work from our research group ([33]). This reference analyzes the use of a pupil in front of an FZP lens. The combination of the FZP lens and the pupil results in a very similar concept as the predistortion technique for the inner regions in the case of maximum predistortion (δ=1). The experimental increase in the focal depth (FLHM) reported in [33] is around 63.0%, while the FWHM slightly decreases, as predicted in our simulations. We have carried out the corresponding simulation with our predistortion technique using the same lens parameters (N=15, L=7, F=270 mm) and operating wavelength (f=250 kHz) as those used in the experiment. We have also used a piston transducer with the same active diameter (D=32 mm) as the experimental ultrasonic source. A piston transducer emitter presents a certain radiation diagram that emphasizes the inner FZP regions over the outer regions and can cause certain deviations between experimental and simulation results, although we have verified that they are not very significant. The piston transducer is separated d=350 mm from the FZP plane. The increase in the focal depth with the predistortion technique is of the order of 83.5% when δ=1. The difference between our simulation result and that of the experiment can be due to several factors. In our opinion, the signal to noise ratio of the measurement presented in reference [33] is not very high, and this could have led to some measurement errors. The lateral resolution is slightly enhanced as the FWHM becomes smaller when predistortion is applied. In this case, we have obtained much closer results between the simulation (FWHM = 87.2%) and the experiment (FWHM = 84.8%). We think that these results are much closer due to the fact that the measurement of the lateral resolution is of much better quality than that of the focusing profile. In any case, we consider that these experimental results demonstrate the feasibility of our predistortion technique and validate both the theoretical analysis and the simulations presented in this work.

## 5. Conclusions

In this work, we have shown that the introduction of controlled errors during the manufacturing stage of an FZP can be used to modified the FZP focusing profile with a certain degree of flexibility. Two different predistortion techniques have been proposed. The first technique deals with predistortion applied to the inner regions. In this case, a number of Fresnel regions, starting with the central region, are conveniently predistorted. It has been shown that, depending on the degree of predistortion applied (δ) and the number of Fresnel regions that are predistorted (*L*), the focal depth (FLHM) of the focusing profile can be significantly increased, while the lateral resolution is slightly improved. The second technique is used when the predistortion is applied to the intermediate Fresnel regions of the FZP. Here, δ and *L* are still the key parameters, but the effect on the FZP focusing profile is completely different, exciting a number of secondary foci adjacent to the main focus. In a way, this effect could still be considered as an enlargement of the main focus, although not as a continuous whole focus as in the case of predistorting the inner regions, but as a discretely distributed larger focus. It has been shown that the main secondary focus can achieve an intensity level as high as 80% of the main focus, although this intensity transfer from one focus to another results in the main focus diminishing its peak intensity value. The focusing profiles that are obtained when intermediate regions are employed present certain similarities with those from Fractal zone plates when Cantor binary sequences are used. Therefore, in all those applications where Fractal zone plates have already succeeded [25,30,31,32], the predistortion technique can be applied to achieve higher flexibility and control.

Summing up, we have introduced three new key parameters in the design of an FZP lens: the degree of predistortion (δ), the number of predistortion regions (*L*), and the location of the predistortion regions inside the FZP (inner regions or intermediate regions). These three parameters can be used together with the conventional design parameters of an FZP lens, such as the number of Fresnel regions (*N*), the location of the main focus (*F*) and the operating frequency (*f*) in order to have a higher degree of flexibility in the design of the FZP focusing profile. We can conclude that predistortion is a useful procedure that introduces additional flexibility in the FZP focusing profile design and can be appealing to research working on a wide range of applications.

## 6. Materials and Methods

Pressure maps and focusing profiles were calculated in MATLAB (The MathWorks Inc., Natick, MA, USA) by numerically computing the Rayleigh–Sommerfeld integral [39].

(7) $$p\left(r,z,\omega \right)=\frac{-jkz}{2\pi }{\;\int \int }_{S}t\left(\rho \right)p_{i}\left(\rho ,\omega \right)\frac{e^{-jkR}}{R^{2}}\rho d\rho d\varphi ,$$

where *S* represents the surface of the lens, $k=\omega /c_{0}$ is the wavenumber, t(ρ) is the transmission function of the lens along its radial axis (ρ), $p_{i}\left(\rho ,\omega \right)$ is the incident pressure at the lens, and $R=\sqrt{z^{2}+r^{2}+\rho^{2}-2r\rho \cos\varphi }$. If ideal spherical wave incidence is considered, the incident pressure is given by

(8) $$p_{i}\left(\rho ,\omega \right)=\frac{e^{-jk\sqrt{\rho^{2}+d^{2}}}}{4\pi \sqrt{\rho^{2}+d^{2}}}.$$

All numerical simulations presented in this work have been carried out considering point source excitation placed at a distance d=350 mm from the FZP plane. The conventional FZP used in this paper has been designed with N=21 Fresnel regions, a main focus at F=50 mm and an operating frequency of f=1 MHz, which corresponds to a wavelength of λ=1.5 mm for underwater transmission. The different predistorted FZPs have been generated from the conventional one, modifying the appropriate radii depending on whether inner or intermediate regions were predistorted, the number of affected regions (*L*), and the degree of the predistortion parameter (δ).

## Data Availability

Data are available upon reasonable request to the corresponding author.
